# First report of bicolour FISH of *Berberis diaphana* and *B. soulieana* reveals interspecific differences and co-localization of (AGGGTTT)_3_ and rDNA 5S in *B. diaphana*

**DOI:** 10.1186/s41065-019-0088-6

**Published:** 2019-04-25

**Authors:** Juncheng Liu, Xiaomei Luo

**Affiliations:** College of Forestry, Sichuan Agricultural University, Huimin Road 211, Wenjiang District, Chengdu City, 611130 China

**Keywords:** (AGGGTTT)_3_, 5S rDNA, FISH, Karyotype, *Berberis diaphana*

## Abstract

**Background:**

*Berberis* consists of approximately 500 species and is the largest genus in Berberidaceae. Most *Berberis* species lack cytological data, and bicolour fluorescence in situ hybridization (FISH) has never been performed on *Berberis*. In this work, a karyotype of *Berberis diaphana*, an alpine *Berberis* species obtained from an altitude of 3600 m in Wolong National Nature Reserve, China, was analysed and compared with *Berberis soulieana* Schneid. via FISH using oligonucleotide telomere probes for (AGGGTTT)_3_ and 5S rDNA (41 bp) for the first time.

**Results:**

*Berberis diaphana* belonged to cytotype 2A and had the karyotype formula 2n = 2x = 28 = 26 m + 2 sm (2SAT). The mitotic metaphase chromosome lengths ranged from 1.82 ± 0.04 μm to 2.75 ± 0.00 μm. Clear (AGGGTTT)_3_ signals were detected at two telomeres in every chromosome and were co–localized with 5S rDNA at the terminal regions of the long arms in the 6^th^ pair of chromosomes. One pair of (AGGGTTT)_3_ sites was localized in the satellites of the 7^th^ pair of chromosomes, which are the only submetacentric chromosomes in this species. Totally 28 chromosomes with one pair of satellited chromosomes were observed in *B. soulieana*. This species had four 5S rDNA signals with two weak signals at the end of long arms in the 5^th^ pair of chromosomes and another two strong signals detected in the interstitial region close to the end of short arms in the 6^th^ pair of chromosomes. Each large signal consisted of two smaller signals with secondary constrictions around them.

**Conclusions:**

FISH physical mapping of *B. diaphana* suggested that (AGGGTTT)_3_ and rDNA 5S co-localize at the 6^th^ pair of chromosomes. The density, location and number difference of 5S rDNA loci indicated structural differences among the chromosomes between *B. diaphana* and *B. soulieana.* Our results provide information that may contribute to future studies on the physical assembly of the *Berberis* genome and the evolution of rDNA and telomere FISH patterns in *Berberis*.

## Background

*Berberis* L. is the largest genus in Berberidaceae and consists of approximately 500 species of evergreen or deciduous simple-leaved shrubs [1, 2]. Many species of this genus are grown as ornamental shrubs and used for medicinal purposes. The whole *Berberis diaphana* Maxim. plant contains berberine [3], which demonstrates anti–neoplastic activities and is used for the treatment of type 2 diabetes [4, 5]. This genus is widely distributed in the temperate and subtropical regions of Asia, Europe, Africa, and North and South America [1] along altitudes ranging from lower than 1000 m to higher than 3000 m [2, 6, 7]. The distribution area has mean annual precipitation ranging from less than 200 to more than 3500 mm [8], thus reflecting the high diversity of their habitats. The stem, leaf, flower, and fruit morphologies of *Berberis* species also demonstrate high diversity [1]. In addition to traditional taxonomy, some molecular studies have been conducted to clarify the phylogenetic relationships and evolutionary history of *Berberis* species [9–12]. However, the possible relationship between the cytogenetic characters and the high diversity of *Berberis* remains uncertain.

Karyotypes have provided many clues for unravelling evolutionary and taxonomic decisions [13]. Chromosome number has been employed in taxometric and cladistic analyses of Berberidaceae and *Berberis* [14, 15]. Wang et al. [15] proposed that Berberidaceae should be divided into three subfamilies (Podophylloideae, Nandinoideae and Berberidoideae) according to the results of chromosome number data, molecular sequence analyses and traditional taxonomic conclusions. Berberidoideae contains *Berberis*, *Mahonia* Nutt and *Ranzania* T. Ito and is the largest subfamily of Berberidaceae. Only three types of chromosome numbers have been observed worldwide in *Berberis*, namely, diploids (2n = 28) [6, 7, 16–18], a few tetraploids (2n = 56) [8, 19] and 2n = 28, 42 in *Berberis amurensis* Rupr. [2]. However, the detailed karyotypes of *Berberis* species have rarely been studied.

Fluorescence in situ hybridization (FISH) can be used to establish detailed karyotypes to reveal inter- and intra-species discrepancies. (TTTAGGG)_n_ originally isolated from *Arabidopsis thaliana* is the telomere sequence for most angiosperms [20]. Lin et al. [21] reported that telomere probes can detect recent centric fusions on muntjacs. 5S rDNA oligonucleotides have been used to identify diversity in the chromosomes of *Piptanthus concolor* [22], and it has also been used to study the genome evolution of another genus of Berberidaceae, *Epimedium* L [23]. Although 45S rDNA FISH has been performed in *Berberis thunbergii* DC [24], thus far, bicolour FISH has not been performed for the karyotype analysis of *Berberis*. In the present study, we analysed the (AGGGTTT)_3_ and 5S rDNA FISH patterns in *B. diaphana* and *B. soulieana* chromosomes in combination with traditional cytological data. Although only two species of *Berberis* were involved in this research, the results will be of great significance in cytogenetic and taxonomic studies for *Berberis* species.

## Results

### FISH with oligo–(AGGGTTT)_3_ and oligo–5S rDNA probes

**Fig. 1:**
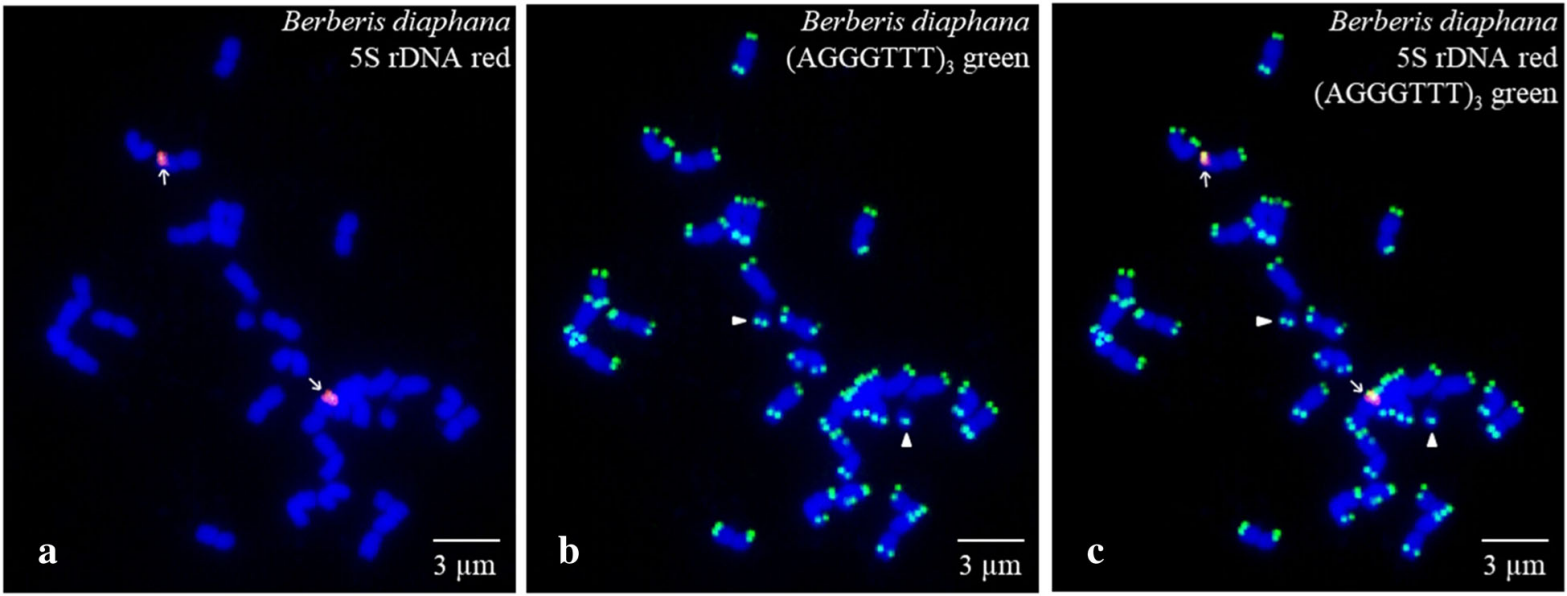
Metaphase plates of *Berberis diaphana* visualized after FISH. Chromosomes probed with 5′– TAMRA–labelled 5S rDNA (red fluorescence, arrow) are shown in **a** and **c**; chromosomes probed with 5′– FAM–labelled (AGGGTTT)_3_ (green fluorescence) are shown in (**b**) and (**c**). The concentration of the probes used for (AGGGTTT)_3_ and 5S rDNA was 10 μM. All chromosomes were counterstained with DAPI (blue). Arrowheads in (**b**) and (**c**) indicate satellites

Mitotic metaphase chromosomes of *B. diaphana* after FISH are shown in Fig. 1. Consistent with the known chromosome number for most *Berberis* species, twenty–eight chromosomes were observed in *B. diaphana*. Only two chromosomes were labelled by 5S rDNA (Fig. 1a and c; red fluorescence, arrow) with relatively strong signals. In terms of the (AGGGTTT)_3_ probe (green fluorescence) shown in Fig. 1b and c, every chromosome displayed strong signals at the terminal regions of the long and short arms, and significant differences were not observed in the densities and locations. However, the satellited chromosome was an exception, and it presented a (AGGGTTT)_3_ probe hybridization site at the terminal region of the long arm and the satellite (arrow head).

**Fig. 2:**
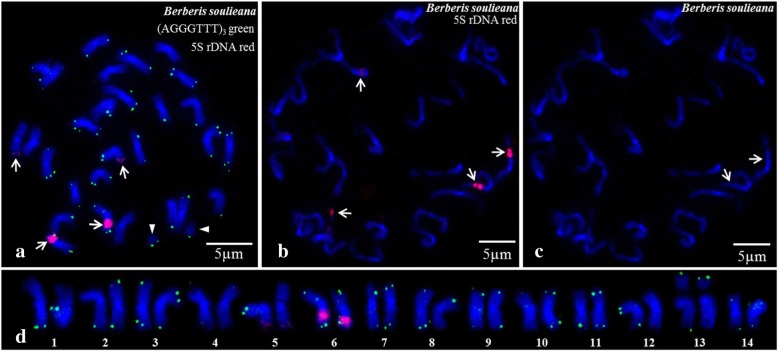
Metaphase (**a**) and prometaphase (**b**, **c**) plates of *B. soulieana* visualized after FISH. In (**a**), (AGGGTTT)_3_ signals are green colour labelled with 5′– FAM; and 5S rDNA signals are red colour labelled with 5′–TAMRA and indicated with arrows, while arrowheads indicate satellites. In (**b**), arrows indicate the 5S rDNA signals of chromosomes. In (**c**), arrows indicate secondary constrictions of the chromosomes with large 5S rDNA signals. Chromosome number in (**d**) of *B. soulieane* was sorted by length (captured from **a**)

Figure 2a shows the FISH results of *B. soulieana*, which are essentially consistent with that of *B. diaphana* in Fig. 1c, showing 28 chromosomes with one pair of satellited chromosomes. The signal of the telomere probe (AGGGTTT)_3_ was also located in the terminal region of each chromosome. However, the location and number of 5S rDNA in *B. soulieana* were different from those of *B. diaphana*. This species had four 5S rDNA signals with two weak signals at the end of long arms in 5^th^ pair of chromosomes and another two strong signals detected in the interstitial region close to the end of long arms in 6^th^ pair of chromosomes (Fig. 2d). In Fig. 2b, each large signal consists of two smaller signals. Moreover, secondary constrictions were found at the gap and the end of two small signals in Fig. 2b and c.

### Karyotype analysis

**Fig. 3:**
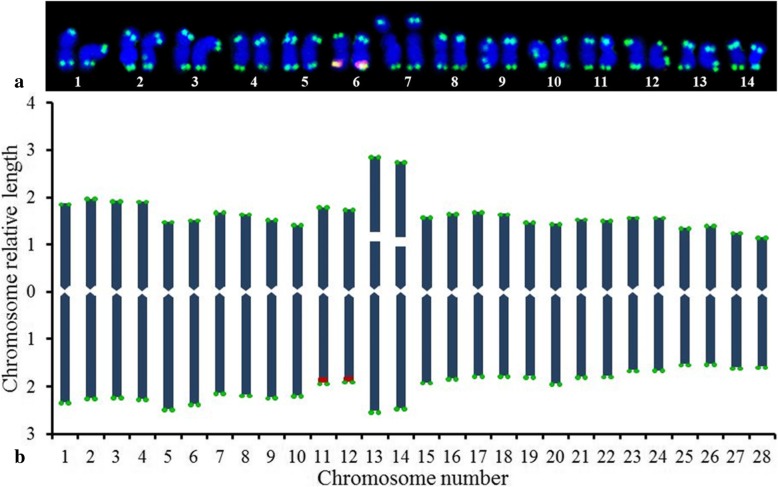
FISH karyotype ideogram summarizing the 5S rDNA (red fluorescence) and (AGGGTTT)_3_ (green fluorescence) signals of *Berberis diaphana*. Chromosome pairs are numbered according to length as measured using NucType version 2013. Satellite length is not included in the chromosome length. Chromosomes in (**a**) were visualized by Photoshop CC 2015 with the images from Fig. 1c. Chromosomes in (**b**) correspond to the chromosome pairs in (**a**). The *x*–axis indicates the chromosome number, and the *y*–axis indicates the relative chromosome length

The karyotype analysis indicated that *B. diaphana* is diploid 2n = 2x = 28 and has a basic chromosome count (x) fourteen. The chromosomes relative lengths (Fig. 3) varied from 4.22 (chromosome 1) to 2.73 (chromosome 28), and the actual lengths for the chromosomes ranged from 2.75 μm to 1.82 μm (Table 1).

**Table 1:** Karyotype and FISH analysis in *B. diaphana*

Chr. No.	Chr. length (μm)			Arm ratio	FISH results			Chr. type
	Long arm	Short arm	L^b^ + S^c^		(AGGGTTT)_3_		5S rDNA	
1	1.50 ± 0.03^a^	1.25 ± 0.03	2.75 ± 0.00	1.20 ± 0.05	L	S	–	m^d^
2	1.47 ± 0.01	1.25 ± 0.01	2.72 ± 0.00	1.17 ± 0.01	L	S	–	m
3	1.58 ± 0.04	0.97 ± 0.01	2.55 ± 0.03	1.62 ± 0.05	L	S	–	m
4	1.41 ± 0.01	1.08 ± 0.01	2.49 ± 0.00	1.31 ± 0.02	L	S	–	m
5	1.44 ± 0.02	0.95 ± 0.04	2.40 ± 0.05	1.51 ± 0.04	L	S	–	m
6	1.25 ± 0.01	1.15 ± 0.02	2.40 ± 0.03	1.09 ± 0.01	L	S	L	m
7	1.63 ± 0.02	0.65 ± 0.04	2.28 ± 0.06	2.49 ± 0.11	L	SA^e^	–	sm^f^
8	1.22 ± 0.03	1.05 ± 0.02	2.28 ± 0.00	1.17 ± 0.05	L	S	–	m
9	1.17 ± 0.00	1.09 ± 0.01	2.25 ± 0.01	1.07 ± 0.01	L	S	–	m
10	1.22 ± 0.05	0.95 ± 0.01	2.17 ± 0.04	1.28 ± 0.07	L	S	–	m
11	1.17 ± 0.00	0.99 ± 0.01	2.16 ± 0.01	1.18 ± 0.01	L	S	–	m
12	1.08 ± 0.00	1.03 ± 0.00	2.11 ± 0.00	1.06 ± 0.01	L	S	–	m
13	1.00 ± 0.00	0.89 ± 0.01	1.89 ± 0.01	1.12 ± 0.02	L	S	–	m
14	1.04 ± 0.01	0.78 ± 0.03	1.82 ± 0.04	1.33 ± 0.04	L	S	–	m

Note: ^a^Mean ± standard deviation, ^b^long arm, ^c^short arm, ^d^metacentric, ^e^satellite, and ^f^submetacentric. The chromosome pair numbers correspond to the numbers in Fig. 2a

Regarding the chromosome length parameters shown in Table 1, the length ratio of the longest/shortest chromosome was 1.48 and the percentage of chromosome-arm ratio more than 2:1 was 0.07. Hence, *B. diaphana* falls into 2A according to the Stebbins karyotype asymmetry degree shown in Table 2. Regarding the type–based chromosome constitution [2n = 2x = 28 = 26 m + 2 sm (2SAT)], *B. diaphana* presented thirteen pairs of metacentric chromosomes and one pair of submetacentric chromosomes, which consisted of two satellited chromosomes (Fig. 3 and Table 1). Due to the centromere and telomere of *B. soulieana* in Fig. 2a are not clear; therefore, the karyotype analysis is not appropriate.

**Table 2:** Karyotype asymmetry index, according to Stebbins (1971)

Chromosome ratio	Percentage (arm ratio > 2:1)			
	0.00	0.01–0.05	0.51–0.99	1.00
<2:1	1A	2A	3A	4A
2:1–4:1	1B	2B	3B	4B
> 4:1	1C	2C	3C	4C

Note: Cytotypes closer to 4C show greater asymmetry

## Discussion

Although 215 *Berberis* species (197 endemic) are found in China, only the chromosome numbers of 5 *Berberis* species have been recorded in *Flora of China* (revised English version): *Berberis ulicina* Hooker et Thomson, 2n = 28 (Page 16); *Berberis julianae* Schneid., 2n = 28 + 2B (Page 26); *Berberis poiretii* Schneid., 2n = 28 (Page 38); *Berberis anhweiensis* Ahrendt, 2n = 28 (Page 47); and *Berberis amurensis* Rupr., 2n = 28, 42 (Page 48) [2]. The *Berberis* chromosome number reported in *Flora of China* (revised English version) is 2n = 14, which is different from the numbers for the species mentioned above; in contrast, in the Chinese version of *Flora of China*, the basic chromosome number of *Berberis* is x = 14 [25]. Although some works have been conducted for chromosome counts, the karyotype analyses of only three *Berberis* species, *Berberis asiatica* Roxb. ex. DC, *Berberis lyceum* Royle [26] and *Berberis julianae* Schneid [16], have been described. Nevertheless, there are few good images of chromosomes in somatic cells, and bicolour FISH has not previously been used to analyse *Berberis* karyotypes.

Consistent with *B. diaphana* and *B. soulieana*, satellited chromosomes have also been reported in other *Berberis* species from eastern Asia, although in some species, satellites were either not observed or the number of satellites was stable at only two [7, 26]. Srivastava et al. [26] showed that the karyotype formulae of *B. asiatica* and *B. lyceum* were 2n = 2x = 28 = 4 m + 22 sm + 2 st and 2n = 2x = 28 = 8 m + 18 sm + 2 st, respectively indicating that the species belong to cytotypes 2B and 1B, respectively. For *B. julianae*, which is also distributed in Southwest China, Huang and Zhao [16] proposed a karyotype formula of 2n = 28 + 2B = 24 m + 4 sm + 2B, indicating that the species belongs to cytotype 2A. In the present study, the karyotype of *B. diaphana* was 2n = 2x = 28 = 26 m + 2 sm (2SAT), indicating that the species belongs to cytotype 2A.

The chromosome sizes for the Indian species *B. asiatica* and *B. lyceum* ranged widely from 3.2 ± 0.07 μm to 11.2 ± 0.09 μm and from 2.1 ± 0.09 μm to 7.33 ± 0.07 μm, respectively [26]. In comparison, the chromosome sizes for *B. diaphana* in this study had a small range of variation from 1.82 ± 0.04 μm to 2.75 ± 0.00 μm (Table 1). Although it is inaccurate to compare the sizes of chromosomes among different species because of variations in cell cycle stages and the degree of squashing, data on ratios, such as the arm ratio and relative length, are still valid [13]. Both *Berberis* species native to Southwest China, i.e., *B. julianae* and *B. diaphana*, exhibited chromosome morphological similarities that were mainly in the identified submetacentric and metacentric chromosomes, and the metacentric chromosomes were dominant. Furthermore, both species belonged to cytotype 2A, which is considered relatively primitive in Stebbins’s system [27]. The two Indian species *B. asiatica* and *B. lyceum* (cytotypes 2B and 1B) have more asymmetric chromosomes than the Chinese species *B. diaphana* and *B. julianae* (cytotype 2A). The major trend in flowering plants is from symmetrical to asymmetrical chromosomes [27]. According to the palaeoenvironment and palaeovegetation study of Sun [28], the ancient altitude of northern Tibet was approximately 2000 m in the Early Miocene, which was suitable for temperate plants to migrate from East Asia to the Indian subcontinent. The discovery of *Berberis* cf. *asiatica* (the conformis of *B. asiatica*) fossils from the Early Miocene indicated that *Berberis* originated in East Asia and then migrated to the Indian subcontinent through northern Tibet. Based on the above discussion, we surmise that the chromosome structure variation from symmetrical to more asymmetrical may have occurred in *Berberis* during the transmission from East Asia to the Indian subcontinent.

This study reports the physical locations of (AGGGTTT)_3_ and 5S rDNA loci as determined by bicolour FISH in *Berberis* species for the first time. The density of the largest signals in *B. soulieana* (Fig. 2a) was much greater than that of *B. diaphana* (Fig. 1a), which suggested that there are more abundant 5S rDNA copies in *B. soulieana*. An increase in the repeat sequences may cause an increase of chromosome length, which could partly explain why the karyotype of *B. diaphana* was quite different from that of the other two Indian *Berberis* species. The secondary constrictions near the largest 5S rDNA signals in the prometaphase chromosomes of *B. soulieana* (Fig. 2b, c) indicated the chromosome structural difference between the two species. The difference in the number and loci of 5S rDNA between *B. diaphana* and *B. soulieana* may be caused by unequal values exchange chromosomes, increased potential rDNA copies by transposons, and chromosome rearrangement [23, 29]. Most species of the genus *Berberis* have 28 chromosomes; hence, a high diversity of this genus may not be due to chromosome number and ploidy. However, revealing the differences in 5S rDNA loci and chromosome structure between *B. soulieana* and *B. diaphana* may provide insights into the crucial role of chromosome structural differences in the diversity of the genus *Berberis*. One pair of 5S signals was localized in the terminal region of the long arms of the chromosomes, whereas in *Epimedium*, another genus of Berberidaceae, one or two pairs of 5S rDNA sites were localized in the interstitial regions of chromosome long arms [23]. Thus, changes in 5S rDNA loci may have occurred between the two genera over the evolutionary course of Berberidaceae as long as the 5S rDNA patterns were concordant among *Berberis* species. Li et al. [30] reported that telomeres and 45S rDNA are co–localized in chromosome telomeric regions in *Chrysanthemum segetum*. Furthermore, telomere and 45S rDNA sequences are structurally linked on the chromosomes of *C. segetum*, although whether such a structural connection between rDNA and telomere sequences occurs in *B. diaphana* remains to be studied.

Bottini et al. [8] indicated that polyploidy may have helped *B. buxifolia* and *B. heterophylla* (2n = 56) adapt to an extremely low–rainfall environment. The results from Meng et al. [31, 32] suggested that polyploids might have played an important role in the evolution of some alpine species. However, studies have indicated that polyploidization may not be a dominant evolutionary process in alpine *Berberis* [7, 18]. Unfortunately, only one of the alpine *Berberis* species in Southwest China was involved in this study. To understand how genetic characteristics affect the adaptability and diversity of *Berberis* species, we will further investigate and compare the cytogenetics of other *Berberis* species via FISH analysis.

## Conclusions

FISH physical mapping of *Berberis diaphana* suggested that (AGGGTTT)_3_ and rDNA 5S co-localize in chromosome pair 6. The density, location and number difference of 5S rDNA loci indicated the structural differences in the chromosomes between *B. diaphana* and *B. soulieana*. Our results provide information that may contribute to future studies on the physical assembly of the *Berberis* genome and the evolution of rDNA and telomere FISH patterns in *Berberis*.

## Methods

### Plant materials and chromosome preparation

*Berberis diaphana* seeds were gathered from an altitude of 3600 m at the Wolong National Nature Reserve, Sichuan Province, China in Oct 2017 then stored at 4 °C, and then they were germinated in soil in May 2018. A *B. soulieana* sapling was obtained from Hubei Province, China. Slide preparation was performed according to the method of Komuro et al. [33] but with modifications. When the root tips reached at 1.5 cm, they were cut and immediately kept in a sealed iron tank full of nitrous oxide for 2–4 h. The root tips were soaked in glacial acetic acid for 15 min and then stored in 75% ethyl alcohol at **−** 20 °C, which can be maintained for long-term periods. We excised 1–2 mm of root tip and placed it in a 0.2 mL tube with 10 μL cellulose and pectinase (2:1) at 37 °C for 1 h. Subsequently, 100 μL dd H_2_O was added to the tube with the root tip and then removed twice, and this step was repeated twice using 100% ethyl alcohol. Then, 20 μL 100% acetic acid was added to each tube and the root tip was stirred into a suspension. A 10 μL drop of the mixture was placed onto a slide, and the air-dried slides were investigated with an Olympus CX21 microscope (Olympus, Japan) and finally stored at − 20 °C after the position of the metaphase cell was marked.

### Probe DNA preparation

The oligogenic sequence repeats probe (AGGGTTT)_3_ consisted of the following 21 bp fragment: 5′ AGGGTTTAGGGTTTAGGGTTT 3′. The 41 bp fragment oligonucleotide 5S rDNA probe 5′ TCAGAACTCCGAAGTTAAGCGTGCTTGGGCGAGGTAGTAC 3′ was reported by Luo et al. [22]. The two oligo-probes were tested in *B. diaphana* and *B. soulieana* and synthesized by Sangon Biotech Co., Ltd. (Shanghai, China). The oligo**–**5S rDNA probe was 5′ end–labelled with 6–carboxytetramethylrhodamine (TAMRA), whereas the (AGGGTTT)_3_ probe was 5′ end–labelled with 6–carboxyfluorescein (FAM). The two oligo**–** probes were dissolved in 1 × TE solution and then stored at − 20 °C with a concentration of 10 μM.

### FISH and karyotype analysis

FISH with bicolour probes was performed as previously described by Luo et al. [22]. The chromosome slides were soaked and fixed in 4% (*w*/*v*) paraformaldehyde for 10 min and then washed twice for 5 min with 2 × SSC. Subsequently, slides were dehydrated with a series of 75, 95, and 100% ethanol for 5 min each and air dried, and then 60 μL of deionized formamide was added to the slides, which were then covered with cover glass. Next, the slides were denatured for 2 min at 80 °C and then immediately placed in an ethanol series at − 20 °C. A 10 μL mixture of 0.35 μL probes, 4.825 μL 2 × SSC and 4.825 μL 1 × TE was dropped onto the air-drying slide, which was then covered with another cover glass. Chromosomes and probes were hybridized at 37 °C on for 1.5 h. The slides were then rinsed twice for 5 min with 2 × SSC and finally with dd H_2_O. The air-dried chromosomes counterstained with 4,6**–**diamidino**–**2**–**phenylindole (DAPI, Vector Laboratories, Inc., Burlingame, USA) were captured with an Olympus BX–63 microscope connected to a Photometric SenSys Olympus DP70 CCD camera (Olympus, Japan).

Raw images were processed with DP Manager Version 3.1.1.208 (Olympus, Japan) and Photoshop CC 2015 (Adobe Systems Incorporated, San Jose, CA, USA). Karyotype data (relative length, arm ratio, and cytotype) were determined with NucType 2013. The actual chromosome lengths were converted from relative lengths based on the real length of the scale bar. The mean actual length and arm ratio values were the average values for homologous chromosomes. Karyotype ideograms were mapped using Excel 2010 based on the relative chromosome lengths. The chromosomes number was determined based on the relative lengths, with the longest assigned the number 1 and the shortest assigned the number 28, and they corresponded to the chromosome pairs 1**–**14. Chromosome classification was performed according to the arm ratio [34] as presented in Table 3, and the cytotype was determined based on the chromosome ratio and the percentage of chromosomes with an arm ratio greater than 2:1 [35] as listed in Table 2. The arm ratio = the length of the long arm/short arm, and the chromosome ratio = the length of the longest chromosome/shortest chromosome.

**Table 3:** Chromosome morphology

Arm ratio	Centromere	Abbreviation
1.00	median point	M
1.01–1.70	median region	m
1.71–3.00	submedian region	sm
3.01–7.00	subterminal region	st
>7.00	terminal region	t
–	terminal point	T

## Abbreviations

5S: 5S rDNA
6-FAM: 6-carboxyfluorescein
6-TAMRA: 6-carboxytetramethylrhodamine
AGT: (AGGGTTT)_3_
DAPI: 4,6-diamidino-2-phenylindole
FISH: Fluorescence in situ hybridization
SSC: Saline sodium citrate
